# Familial Hypercholesterolemia Prevalence Among Ethnicities—Systematic Review and Meta-Analysis

**DOI:** 10.3389/fgene.2022.840797

**Published:** 2022-02-03

**Authors:** Frida Toft-Nielsen, Frida Emanuelsson, Marianne Benn

**Affiliations:** ^1^ Department of Clinical Biochemistry, Rigshospitalet, Copenhagen University Hospital, Copenhagen, Denmark; ^2^ Department of Clinical Medicine, Faculty of Health Sciences, University of Copenhagen, Copenhagen, Denmark

**Keywords:** familial hypercholesterolemia, ethnicity, race, epidemiology, general population

## Abstract

**Background:** Heterozygous familial hypercholesterolemia (FH) is a common genetic disorder leading to premature cardiovascular disease and death as a result of lifelong high plasma low-density lipoprotein cholesterol levels, if not treated early in life. The prevalence of FH varies between countries because of founder effects, use of different diagnostic criteria, and screening strategies. However, little is known about differences in FH prevalence according to ethnicity. We aimed to investigate the ethnic distribution of FH in diverse populations and estimate the prevalence of FH according to ethnicity.

**Methods:** We performed a systematic review and meta-analysis, searching PubMed and Web of Science for studies presenting data on the prevalence of heterozygous FH among different ethnicities in non-founder populations. Studies with more than 100 individuals, relevant data on prevalence, ethnicity, and using the Dutch Lipid Clinical Network Criteria, Simon Broome, Making Early Diagnosis Prevents Early Death, genetic screening, or comparable diagnostic criteria were considered eligible for inclusion.

**Results:** Eleven general population studies and two patient studies were included in a systematic review and 11 general population studies in a random-effects meta-analysis. The overall pooled FH prevalence was 0.33% or 1:303 in 1,169,879 individuals (95% confidence interval: 0.26–0:40%; 1:385–1:250). Included studies presented data on six ethnicities: black, Latino, white, Asian, brown, and mixed/other. Pooled prevalence was estimated for each group. The highest prevalence observed was 0.52% or 1:192 among blacks (0.34–0.69%; 1:294–1:145) and 0.48% or 1:208 among browns (0.31–0.74%; 1:323–1:135) while the lowest pooled prevalence was 0.25% or 1:400 among Asians (0.15–0.35; 1:500–1:286). The prevalence was 0.37% or 1:270 among Latino (0.24–0.69%; 1:417–1:145), 0.31% or 1:323 among white (0.24–0.41%; 1:417–1:244), and 0.32% or 1:313 among mixed/other individuals (0.13–0.52%; 1:769–1:192).

**Conclusion:** The estimated FH prevalence displays a variation across ethnicity, ranging from 0.25% (1:400) to 0.52% (1:192), with the highest prevalence seen among the black and brown and the lowest among the Asian individuals. The differences observed suggest that targeted screening among subpopulations may increase the identification of cases and thus the opportunity for prevention.

## Introduction

Familial hypercholesterolemia (FH) is a genetic disorder of lipoprotein metabolism, known to increase levels of total and low-density lipoprotein (LDL) cholesterol in plasma. FH is caused by various mutations, with the majority of known mutations affecting the LDL receptor, apolipoprotein B, and proprotein convertase subtilisin/kexin 9 (Rader et al., 2003; Austin et al., 2004). Without treatment, the lifelong increased LDL-cholesterol levels result in a high risk of premature atherosclerotic cardiovascular disease and death (Nordestgaard et al., 2013a). The diagnosis of FH is confirmed either by applying one of the several clinical criteria, the most common being Dutch Lipid Clinical Network Criteria (DLCN), Simon Broome (SB), and Making Early Diagnosis Prevents Early Death (MEDPED), or by genetic screening.

In a general population setting, FH has recently been estimated to affect 1:313 individuals worldwide, making FH one of the most common genetic disorders in the world (Beheshti et al., 2020). In a Danish study investigating the general population, the estimated prevalence was 1:137 (Benn et al., 2012), suggesting that, in a general population setting, FH is underdiagnosed, emphasizing the importance of efficient FH screening to identify individuals at risk (Beheshti et al., 2020). Identification of subgroups at high risk of FH may facilitate a targeted screening worldwide.

Recent studies have shown that the prevalence of FH varies between countries because of founder effects, use of different diagnostic criteria, and differences in screening for the disease (Beheshti et al., 2020), (Hu et al., 2020)^.^ However, no previous study summarized differences in the prevalence of FH among ethnic groups (Harada et al., 2018).

We performed a systematic review and meta-analysis to examine the prevalence of FH among different ethnicities.

## Methods

### Search Strategy and Screening Process

**FIGURE 1:**
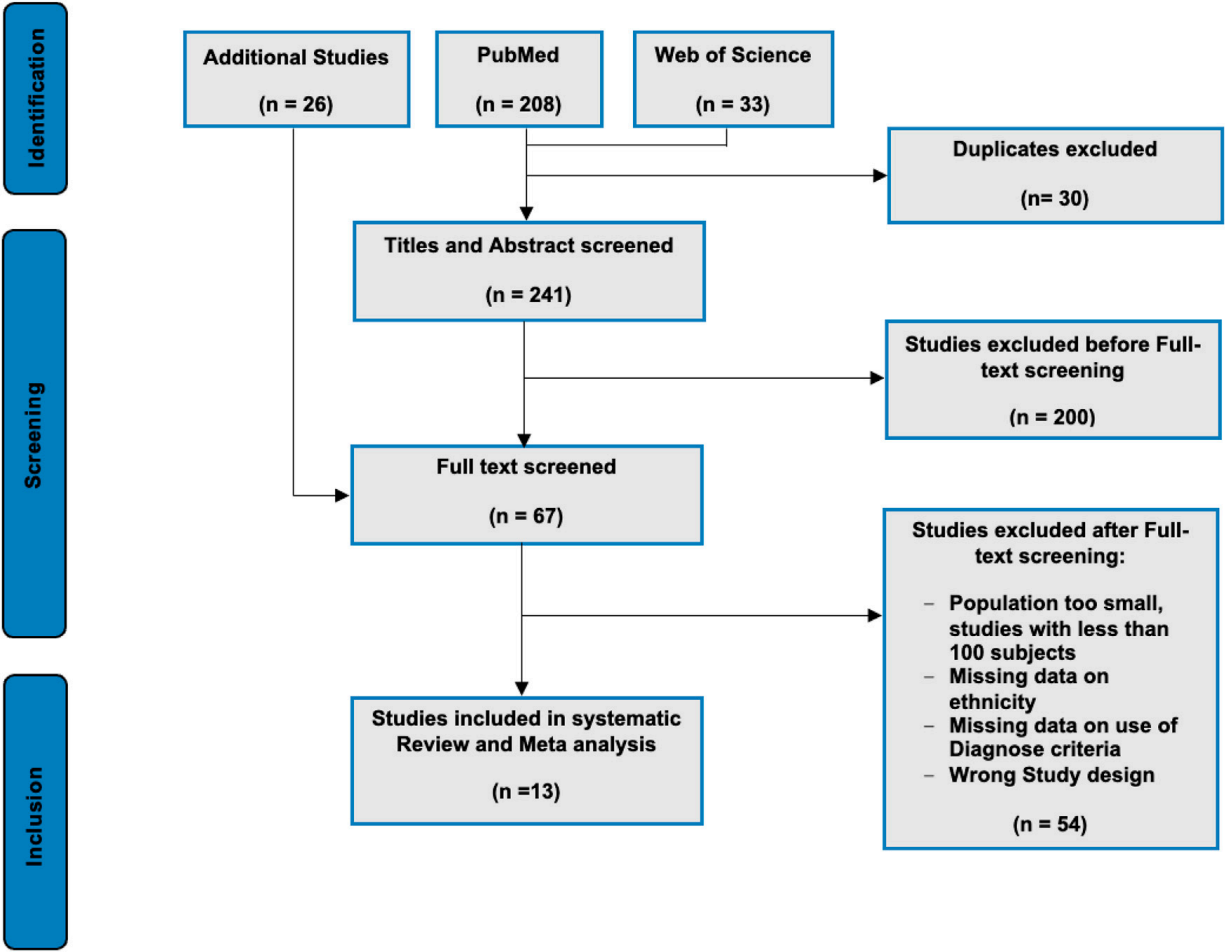
PRISMA flowchart of the inclusion of studies for the review and meta-analysis.

PubMed and Web of Science were searched for possible eligible studies. The last search was made on 30 December 2021. The following MeSH terms were used to search the databases: “Familial Hypercholesterolemia,” “Prevalence,” and “Ethnicity.” In the Web of Science database, two separate search strategies were used: “Familial Hypercholesterolemia,” “Prevalence,” and “Ethnicity”; and “Familial Hypercholesterolemia,” “Prevalence,” and “Ethnic groups” (Figure 1).

Data were managed in the systematic review software Covidence (Covidence, 2021). Duplicates were removed, and the studies were initially screened based on the study’s title/abstract, and irrelevant publications were excluded (Figure 1). Systematic reviews and meta-analyses were excluded, and only full-research articles were included for further screening. The remaining publications were subsequently full-text-screened. Additionally, we screened the reference lists from excluded articles/studies with relevant data, and qualified studies were added directly to the full-text screening.

Studies were defined as eligible if they met any of the following pre-defined inclusion criteria:

1) The study cohort: cohorts representative of the general population and patient cohorts with more than one ethnicity were defined as eligible.
2) The size of the study cohort: only studies of a minimum of 100 subjects were included.
3) The use of the following diagnostic criteria: DLCN, SB, MEDPED, genetic screening, or similar criteria where all individuals in the study were diagnosed using the same criteria.
4) Information on ethnicity.
5) Reporting FH prevalence: the prevalence of individuals diagnosed with FH in the full sample and among the different ethnicities was either reported or could be calculated from the available data.
6) Language: only English language studies were included.

Studies that did not meet the inclusion criteria or that met any of the following exclusion criteria were excluded:

1) Deficient data on the ethnic distribution in the study cohort or unclear definition of the diagnostic criteria used.
2) Studies with FH screening of populations with known founder mutations because the FH prevalence in these populations is known to be higher among specific subpopulations.

In cases where more than one study used the same cohort, we included the publication with the most available data on ethnicity and FH prevalence. An attempt was made to contact authors if relevant studies were lacking important data.

The included studies were divided into two categories: “general population” and “patient cohorts.” Studies were categorized as patient studies if the subjects in the cohort were included from lipid clinics and hospitals or if the subjects were included based on elevated lipid levels or premature coronary artery disease. Studies with subjects included from the general population, stated by the authors, were categorized as general population studies. The following data were extracted: author, publication year, country, sex, mean age, diagnostic criteria used, total number of subjects, overall FH prevalence, the ethnic distribution of the full study cohort (black, Latino, white, brown, Asian, mixed/other), the prevalence of FH among ethnicities (Table 1).

**TABLE 1:** Characteristics of all studies included in the systematic review and meta-analysis.

Studies including more than one ethnicity													
Study	Author	Year	Country	Source	Mean age	Sex, %	FH criteria		Sample size, N	Total FH cases, N	Total prevalence, %	Ethnicities	Prevalence and ethnicity %
NHANES de Ferranti et al. (2016)	De Ferranti et al.	2016	United States	GP	46.8 years	F: 51.4	DLCN		36,949	148	0.4	Black, Latino, white, mixed/other	Black: 0.46 Latino: 0.37 white: 0.40 mix./oth.: 0.28
						M: 48.6									
ELSA-Brasil Harada et al. (2018)	Harada et al.	2018	Brasil	GP	HeFH: 55 years	F: 54	DLCN		14,460	55	0.38	16% black, 53% white, 29% mixed	Black: 0.67, white: 0.25, brown: 0.48
						M: 46									
The Cape Town Experience Firth and Marais (2008)	Firth et al.	2008	South Africa (Cape Town)	Patients from a lipid clinic	HeFH: 44 years	F: 53	Clinical equal to modified DLCN		4,494	1,029	23	Black, white, Asian colored	Black: 4.90, white: 32.2, Asian: 19.8 mix./oth.: 17.2
						M: 47									
MyHEBAT FH Study, Malaysia Chua et al. (2021)	Chua et al.	2021	Malaysia	GP	53.7 years	F: 62.6	DLCN		5,135	55	1.1	Malay, Chinese, Indian, Others	Asian: 1.2%, Others: 0.4%
						M: 38.4									
The Young MI Registry Singh et al. (2019)	Singh et al.	2019	United States	Young adults with MI	45 years	F: 19.1	DLCN		1,996	180	9	Black, hispanic/Latino, white, Asian, mixed/other	Black: 9.8, Latino: 14.0.98 white: 8.79, Asian: 4.3 mix./oth. : 8.1
						M: 80.9									
Studies including one ethnicity													
Copenhagen City Heart Study, DK Tybjaerg-Hansen et al. (2005)	Tybjærg-Hansen et al.	2005	Denmark	GP	53 years	F: 55		DLCN	9,255	15	0.17	100% white	White: 0.17
						M: 45							
Copenhagen General Population Study Benn et al. (2016)	Benn et al.	2016	Denmark	GP	NA	F: 55		Genetic	98,098	450	0.46	100% white	White: 0.46
						M: 45							
LIFE Child Health Cohort, GE Dathan-Stumpf et al. (2016)	Dathna-Stumpf et al.	2016	Germany	GP	0–16 years	F:52.3		Clinical HeFH	2,571	6	0.23	Caucasian	White: 0.23
						M: 47.7							
MyCode cohort, US Abul-Husn et al. (2016)	Abul-Husn et al.	2016	United States	GP	61 years	F: 59.2, M: 40.8		Genetic	43,979	172	0.39	98.4% Caucasian	White: 0.39
Allina Health ambulatory Facility, US Knickelbine et al. (2016)	Knickelbine et al.	2016	United States	GP	FH:53 No FH: 54.1 years	F: 55		Clinical National Lipid Assoc. Guidelines	391,166	841	0.21	90% white	White: 0.21
						M: 45							
Jiangsu Nutrition Study Wijesekera et al. (2014)	Shi et al.	2014	China	GP	57 years	NA		DLCN	9,324	26	0.28	Asian	Asian: 0.28
Kumamoto Health Care, Japan Ohta et al. (2002)	Ohta et al.	2002	Japan	GP	18 months	NA		Clinical HeFH family study	56,181 adj. size: 47,877	91	0.19	Asian	Asian: 0.19
Korean Meta. Syndrome Mortality Study Jung et al. (2018)	Jung et al.	2018	Korea	GP	44.3 years	F: 42.6		MEDPED	502,966	540	0.11	Asian	Asian: 0.19
						M: 57.4							

GP, general population; F, female; M, male; HeFH, heterozygous familial hypercholesterolemia; DLCN, Dutch Lipid Clinic Network Criteria; MEDPED, Make Early Diagnosis Prevent Early Death; NA, not available.

### Diagnostic Criteria for Familial Hypercholesterolemia

Studies classifying FH according to either DLCN, SB, MEDPED, similar clinical criteria, or genetic examination were included. The DLCN determines the likelihood of an individual having FH based on family history, own clinical history, a physical examination, LDL-cholesterol concentration, and a genetic examination. Depending on the total score, individuals are categorized into having unlikely FH (score <3), possible FH (score 3–5), probable FH (score 6–8), or definite FH (score ≥8). In the present review and meta-analysis, we considered individuals with probable and definite FH (score ≥6) as having FH (Nordestgaard et al., 2013a) (Supplementary Table S1).

The SB criteria predict the risk of an individual having FH based on clinical and genetic factors and family history (Supplementary Table S2). MEDPED determines the probability of a patient having FH based on age, family history, and total cholesterol levels (Birnbaum et al., 2021) (Supplementary Table S3).

### Ethnicity

In order to compare prevalence among ethnicities, the following six groups were defined: black, Latino, white, brown, Asian, mixed/other, on behalf of the available data. The ethnicities presented in each study were then allocated into one of the defined groups.

### Prevalence

Data were summarized using prevalence estimates to facilitate the comparison between different ethnicities. If the prevalence was not directly available for data extraction, it was calculated from the size of the total study cohort, the number of individuals with FH, and ethnic distribution in the study. Confidence intervals for prevalence were calculated by score (Wilson) (Nyaga et al., 2014).

### Statistical Analyses

We used StataSE 16.1. to examine differences in prevalence among ethnicities and the *metaprop* command to estimate the prevalence of studies combined (Nyaga et al., 2014). Two-sided *p*-values for the difference between the overall population prevalence compared to the prevalence in each ethnic group were calculated using the *prtest* of proportions in Stata, where *p*-values < 0.05 were considered statistically significant (Acock, 2018). In the meta-analysis, between-study heterogeneity was assessed by I (Rader et al., 2003) statistics (Higgins et al., 2003). A random-effect model was chosen to accommodate potential between-study heterogeneity due to the inclusion of studies using different inclusion criteria.

## Results

Of the 267 screened publications, a total of 13 studies, comprising 1,175,249 individuals, were included (Figure 1). Characteristics of the included studies are shown in Table 1. Five studies reported FH prevalence among more than one ethnicity, whereas the remaining eight studies reported FH prevalence in one specific ethnicity (Table 1). The included studies originated from North America (four studies from the United States), Asia (four studies from China, Malaysia, Japan, and Korea), Europe (two studies from Denmark and Germany), South America (Brazil), and South Africa (Cape Town).

Studies included in the meta-analysis utilized both clinical and genetic screening to estimate FH prevalence. Seven studies applied the DLCN (or modified versions), one study applied the MEDPED, two studies applied genetic screening, and the remaining three studies applied another clinical screening method. Studies included were published between 2002 and 2019.

Eleven studies reported the prevalence of FH in general populations, including information on ethnicity, while two studies estimated the FH prevalence across ethnicity in patient cohorts (Table 1).

The following studies estimated the FH prevalence in more than one ethnicity: NHANES, the ELSA-Brasil study, Cape Town Experience, MyHEBAT FH study, and the YOUNG-MI Registry. NHANES represented the ethnicities: non-Hispanic black (considered as black individuals), non-Hispanic white (considered as white individuals), other race/multiracial (considered as mixed/other individuals), Mexican American, and other Hispanics (considered as Latino). The ELSA-Brasil study reported on the following ethnicities: black, white, Asian, and brown, whereas the MyHEBAT study represented Malay, Chinese, and Indian individuals (all considered Asian) and other individuals (de Ferranti et al., 2016; Harada et al., 2018; Chua et al., 2021).

The two patient cohort studies reported on the following ethnicities: black, white, Asian, and colored (considered as mixed race/other) in the Cape Town Experience; and black, Hispanic/Latino (considered as Latino), white, Asian, and mixed/other (considered as mixed race/other) in the YOUNG -MI Registry (Firth and Marais, 2008; Singh et al., 2019).

The remaining eight studies included only one ethnicity: the Copenhagen City Heart Study and the Copenhagen General Population Study with 100% white individuals, the Life-Child cohort reported all as being Caucasian, My Code Cohort as 98.4% Caucasian, and the Alina Health ambulatory as 90% white (Tybjaerg-Hansen et al., 2005; Abul-Husn et al., 2016; Benn et al., 2016; Dathan-Stumpf et al., 2016; Knickelbine et al., 2016). These individuals were all considered white. Individuals included in the Jiangsu Nutrition Study, Kumamoto Health Care, and Korean Metabolic Syndrome Mortality Study were considered Asian (Ohta et al., 2002; Wijesekera et al., 2014; Jung et al., 2018).

### Prevalence of FH in Studies Including More Than One Ethnicity

**FIGURE 2:**
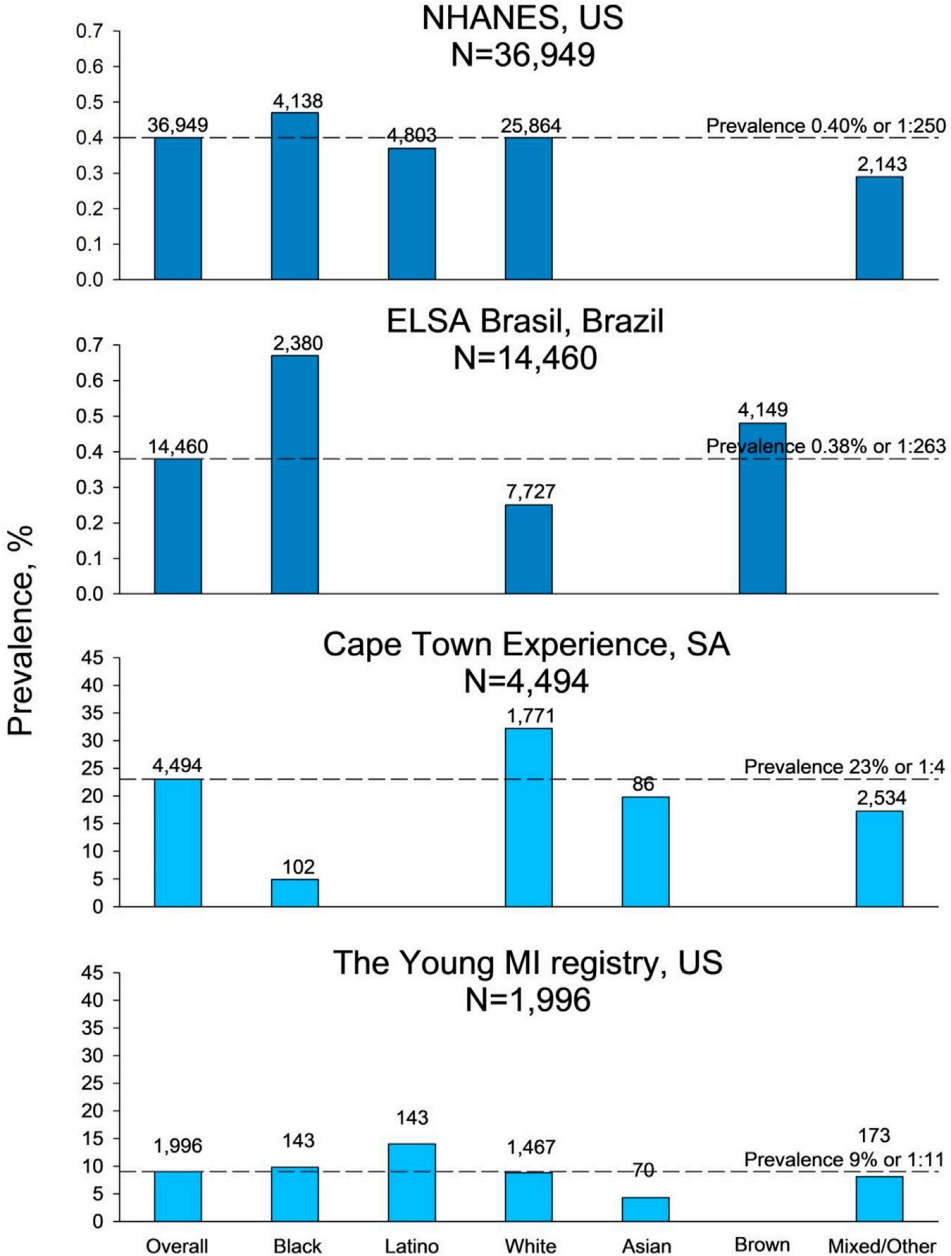
Prevalence of familial hypercholesterolemia in studies including more than two ethnicities. Prevalence shown with dark blue bars is from general population studies, and prevalence shown with light blue bars is from patient cohort studies. For information on ethnicity, refer to Methods. N, number of participants in the study, US, United States of America, SA, South Africa.

Of the five included studies reporting FH prevalence among more than one ethnicity, three studies were performed in a general population setting and two studies in patient cohorts (Table 1). In the NHANES, the prevalence was 0.40% or 1:250 and in the ELSA-Brasil, 0.38% or 1:263 (Figure 2). In both NHANES and ELSA-Brasil, the prevalence of FH was high among black individuals compared to the overall population prevalence, with a prevalence in black of 0.46% or 1:249 in NHANES and 0.64% or 1:156 in ELSA-Brasil. In ELSA-Brasil, the prevalence was also higher for individuals identified as brown: 0.48% or 1:208, compared to the overall population prevalence. In NHANES, individuals identified as mixed/other had a lower prevalence than the overall population: 0.28% or 1:357, while in ELSA-Brasil, individuals identified as white had a lower FH prevalence of 0.25% or 1:417 compared to the overall population prevalence. In the MyHEBAT study, including Asian and mixed/other individuals, the overall FH prevalence was 1.1% or 1:100, with a prevalence of 1.2% or 1:83 in Asian individuals and of 0.4% or 1:250 in mixed/other individuals.

In the patient cohort studies, the overall reported prevalence of FH was 23% or 1:4 in the Cape Town Experience and 9% or 1:11 in the YOUNG-MI Registry (Figure 2). In the Cape Town Experience, the estimated prevalence among the white individuals of 32% or 1:3 was high compared to the overall population prevalence. In comparison, the prevalence in individuals identified as black (4.9% or 1:20), Asian (20% or 1:5), and mixed/other (17% or 1:6) had a lower prevalence compared to the overall study prevalence. In the YOUNG-MI Registry, individuals identified as Latino (14% or 1:7) had a high prevalence compared to the overall FH prevalence, while Asian individuals had a low prevalence of 4.3% or 1:23 compared to the overall FH prevalence.

### Pooled Prevalence of FH in General Population Studies

**FIGURE 3:**
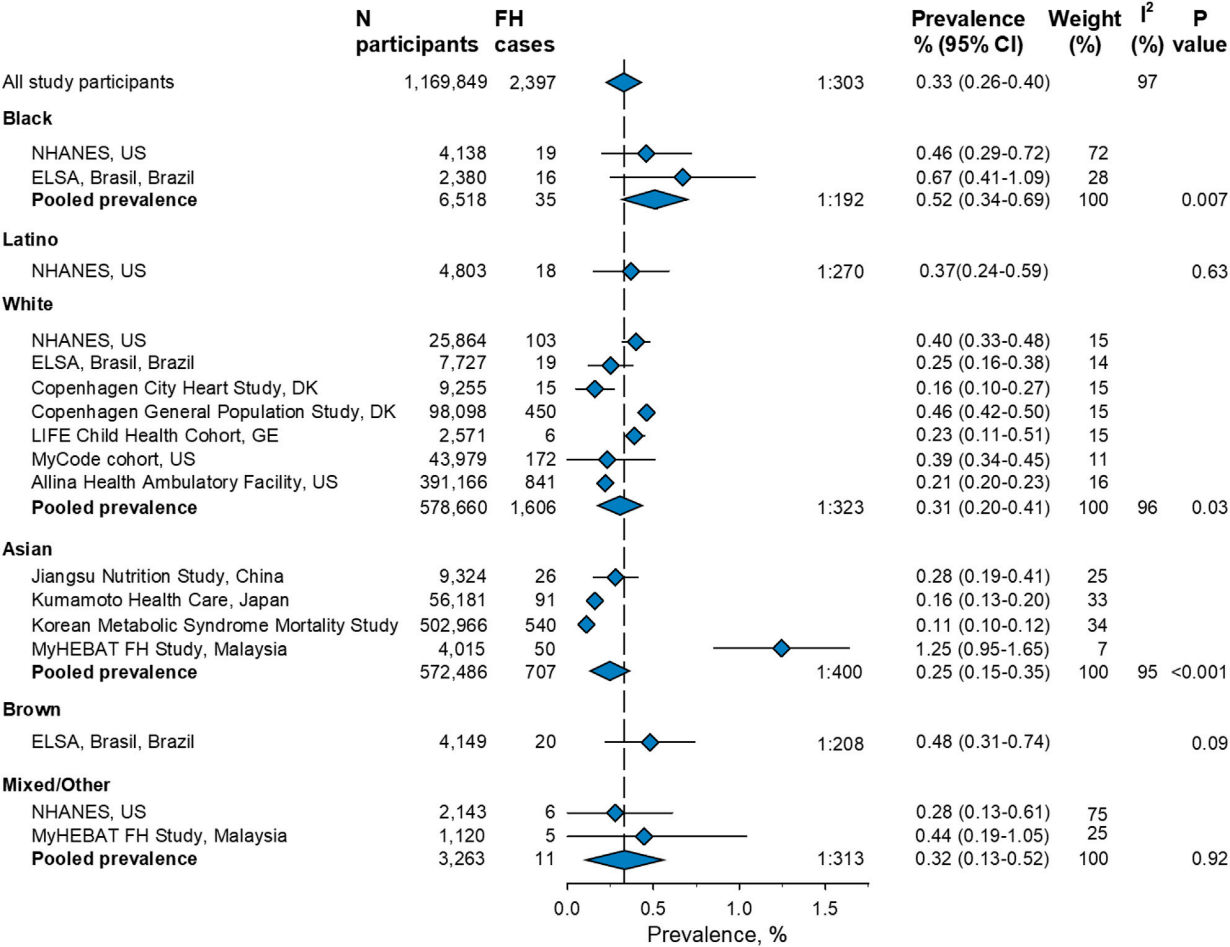
Prevalence of familial hypercholesterolemia among ethnicities in 11 general population studies included in a meta-analysis. Small diamonds represent point estimates for individual studies. Large diamonds represent pooled meta-analysis estimates. Weight (%) is the weight of the study within each meta-analysis. Test for heterogeneity [I (Rader et al., 2003)] was significant for all meta-analyses (all *p* < 0.001). *p*-value is for comparison of the estimates for each ethnicity compared to the overall estimate (represented by the vertical broken line) of all participants. For information on ethnicity, refer to Methods. N, number, FH, familial hypercholesterolemia, CI, confidence interval.

Eleven studies were included in the meta-analysis of general population studies, and a pooled prevalence for each of the six ethnicities (black, Latino, white, Asian, brown, and mixed/other) was calculated (Figure 3). The overall FH prevalence in the 1,169,879 individuals from the 11 general population studies was 0.33% or 1:303 (95% confidence interval: 0.26–0:40%; 1:385–1:250). The pooled FH prevalence according to ethnicity ranged from 0.25% or 1:400 in Asian individuals to 0.52% or 1:192 in black individuals. The prevalence of 0.52% or 1:192 (0.34–0.69; 1:294–1:145) in black individuals was higher compared to the overall FH prevalence (*p* = 0.007), while the prevalence of 0.31% or 1:323 (0.24–0.41; 1:417–1:244) in white individuals and 0.25% or 1:400 (0.15–0.35; 1:500–1:286) in Asian individuals was lower compared to the overall FH prevalence (*p* = 0.03 and *p* < 0.001, respectively). The pooled prevalence of 0.37% or 1:270 (0.24–0.69; 1:417–1:145) in Latinos, 0.48% or 1:208 (0.31–0.74; 1:323–1:135) in brown individuals, and 0.32% or 1:313 (0.13–0.52%; 1:769–1:192) in individuals defined as mixed/other did not differ significantly from the overall prevalence (all *p* > 0.09).

## Discussion

In the current study, we estimated the overall FH prevalence to 1:303 in the general population. This is very similar to recent estimations presented in larger studies of 1:313 (Beheshti et al., 2020) and 1:311 (Hu et al., 2020). The pooled prevalence by ethnicity ranged from 0.25% (1:400) to 0.52% (1:192), showing an increase in prevalence from Asian to white to brown to black, suggesting that prevalence differs among ethnicities and that some ethnic groups have a higher risk of FH.

Studies included in the meta-analysis utilized both clinical and genetic screening to estimate FH prevalence. Seven studies applied the DLCN (or a modified version), one study the MEDPED, two studies genetic screening, and the remaining three studies another clinical screening method. The use of clinical screening has several advantages. It may be preferable in areas with limited access to genetic testing and healthcare facilities and may easily be applied in large general population studies, as information about premature coronary artery disease, family history, and clinical measurements is readily obtainable. However, the use of clinical screening may result in an overestimation of FH prevalence, as individuals with other types of dyslipidemias or high lipoprotein (a) may score high in the diagnostic criteria without having genetic FH, resulting in false positives (Hu et al., 2020) although these individuals are at high risk of cardiovascular disease. Similarly, common confounding cardiovascular risk factors such as obesity, diabetes, hypothyroidism, and a high alcohol intake may all cause dyslipidemia, also resulting in false-positive cases and an overestimation of FH prevalence (Nordestgaard et al., 2013b). Differences in these common cardiovascular risk factors together with differences in access to appropriate health care may also explain the differences in the prevalence of FH observed in the present study, although we cannot present data to support this.

As also shown in other studies, the prevalence of FH is significantly higher among patients with premature coronary artery disease and elevated cholesterol concentrations compared to the prevalence observed in general population studies (Beheshti et al., 2020; Hu et al., 2020). In the daily clinic cascade, testing of first-degree relatives is recommended, and it has been suggested that an approach combining contact to relatives through index cases (indirect testing) and direct contact from health care professionals to the relatives (direct testing) may improve the proportion of tested individuals. We did not have information on screening strategy in the patient cohorts included in the present review. However, the choice of strategy may have biased prevalence estimates among ethnicities within the single study (Leonardi-Bee et al., 2021).

The clinical criteria used in FH studies are designed primarily to detect FH in Western populations, and the DLCN, SB, and MEDPED might be less applicable in other populations. This may affect the validity of estimated prevalence in these countries and impact the diagnostic validity in different populations and among ethnicities (Hu et al., 2020).

Genetic screening is considered the most accurate method for diagnosing FH, as the detection of an established disease-causing mutation equals a definite FH diagnosis (Cuchel et al., 2014). Genetic screening is most often applied in patient cohorts to verify the cause of dyslipidemia or establish the presence of a mutation in cascade screening. However, genetic screening is not without limitations. It is time-consuming, is costly, and requires access to health facilities, making it less applicable in less affluent parts of the world (Hu et al., 2020). Moreover, studies using genetic screening report low prevalence estimates of FH, which may be due to not all FH-causing mutations having been identified or included in diagnostic testing panels for FH. Some individuals may present with a polygenic rather than a monogenic cause of FH, which is not always detected (Nordestgaard et al., 2013b; Talmud et al., 2013). In addition, genetic screening often requires evidence from functional studies for further classification of the associated variant as pathogenic or likely pathogenic (Chora et al., 2018). Furthermore, functional characterization of the variant is important for choosing the most effective treatment strategy (Di Costanzo et al., 2021).

In the present study, the estimated prevalence is the highest among black individuals: 0.52% (1:192). However, white individuals had an estimated prevalence of 0.31% (1:323), which is close to the overall estimate. In contrast to this, the prevalence by ethnicity reported in the patient cohort the Cape Town Experience (Firth and Marais, 2008), where 32% of the FH patients were white and 4.9% were black, suggest a bias in access to study participation, potential dissimilarities, or bias in screening strategies.

To successfully investigate differences in prevalence of FH among different ethnicities, a definition of ethnicity was needed. In the included studies, the ancestry of the individuals was either described through race or ethnicity. In four out of five studies representing more than one ethnicity, both terms were used (de Ferranti et al., 2016; Harada et al., 2018). The two concepts are both related to the ancestry of individuals, as race is often described as the distinctive physical trait of an individual, that is, the biology, while the ethnicity is more individual or subjective and is acquired through a cultural identification of the individual. Both terms are considered social concepts, meaning that there are no links between genes and ethnicity. In the present study, the categories of ethnicities were given and limited by the studies included.

The major limitations of the present study are the limited availability of data on prevalence among ethnicities and heterogeneity in diagnostic criteria used. A very limited number of studies reported data on the distribution of FH across ethnicities. White and black individuals were included in most studies, while the inclusion of other ethnicities was limited, potentially introducing bias of estimates of prevalence among less represented ethnicities. Only one study reported FH prevalence among Latino and brown individuals, respectively, and a pooled prevalence could not be estimated for these subgroups.

The 13 studies included used different diagnostic criteria, which might bias and contribute to the heterogeneity of the estimated pooled prevalence. Four out of seven studies applying DLCN used a modified version of the screening method (Wijesekera et al., 2014; Benn et al., 2016; de Ferranti et al., 2016; Harada et al., 2018). The modifications made were due to a lack of data, such as missing information on xanthomas and corneal arcus, or insufficient information on family history. These limitations might have led to an underestimation of the FH prevalence in these studies (Wijesekera et al., 2014; Benn et al., 2016; de Ferranti et al., 2016; Harada et al., 2018). However, minor modifications of the criteria were accepted, and the given studies were still considered eligible for inclusion.

The studies included represent different cohorts, with variations in sex distribution and mean age. These differences might further contribute to between-study heterogeneity. Two out of the 11 general population studies examined the FH prevalence in pediatric cohorts (Ohta et al., 2002; Abul-Husn et al., 2016). Clinical screening of FH among children may have to be interpreted differently compared to the results presented in studies examining adult populations. Children might present with lower LDL-cholesterol levels than adults, and a family history of premature myocardial infarction may not be available, as the parents of younger children may be too young to have signs of myocardial infarction. These factors may contribute to underestimating FH prevalence (Pang et al., 2016). However, the prevalence estimated in the two pediatric cohorts was similar to other FH prevalence estimates presented within the ethnic group.

Another limitation might be the variations in the representation of men and women within the different study cohorts. The YOUNG-MI Registry cohort consisted of 80.9% men compared to 19.1% women (Singh et al., 2019). This underrepresentation of women might affect the estimated FH prevalence, especially when clinical screening methods, such as DLCN, are applied. The DLCN includes own myocardial infarction as a criterion, and as women in general have myocardial infarction at an older age compared to men, an underestimation of FH prevalence may be seen in cohorts with less women compared to men. This could also explain the differences seen among the two included patient studies, as the estimated FH prevalence in the YOUNG-MI Registry is significantly lower than the FH prevalence measured in the Cape Town Experience (Firth and Marais, 2008).

Robust examination of prevalence by ethnicity requires large studies with sufficient data on both FH prevalence in the entire study cohort and among ethnicities. In the present study, study cohorts originating from different countries have been included. For future studies, the ideal setting would be to investigate the prevalence of FH by ethnicity within one country, representing an admixed and diverse society but within the same healthcare system and using the same clinical FH criteria. Focus on aligning sex and age to minimize study heterogeneity may also be important. This set-up would reduce potential bias and give an opportunity to further explore potential differences in FH prevalence according to ethnicity and potential inequalities in access to and participation in screening programs, helping to guide targeted screening and prevention.

In this systematic review and meta-analysis, we found that FH prevalence varies across different ethnicities, ranging from 0.25% (1:400) to 0.52% (1:192), with the highest prevalence seen among black and brown individuals and the lowest prevalence estimated among Asian individuals. There is a need for studies investigating FH prevalence according to ethnicity to establish potential benefits of intensified FH screening in certain population subgroups and further explore potential biases and inequalities in current FH diagnostic criteria and screening programs.

## Data Availability

The original contributions presented in the study are included in the article/Supplementary Material, Further inquiries can be directed to the corresponding author.
