# Supplementary material for: Familial Hypercholesterolemia Prevalence Among Ethnicities—Systematic Review and Meta-Analysis
Source: Front Genet. 2022 Feb 3;13:840797. doi: 10.3389/fgene.2022.840797 (PMC8850281; doi:10.3389/fgene.2022.840797)
Supplement: Supplementary file 1 [file DataSheet1.PDF]

Supplementary data

Familial Hypercholesterolemia prevalence among ethnicities – systematic review and meta-analysis

Frida Toft-Nielsen, BSci[1,2], Frida Emanuelsson, MD, PhD[1], Marianne Benn, MD, PhD, DMSc[1,2].

[1]Department of Clinical Biochemistry, Rigshospitalet, Copenhagen University Hospital, Denmark; [2]Faculty of Health Sciences, Department of Clinical Medicine, University of Copenhagen, Denmark.

Running head: Familial hypercholesterolemia and ethnicity

Corresponding author:

Marianne Benn, professor, chief physician, MD, PhD, DMSc.

Department of Clinical Biochemistry

Rigshospitalet, Copenhagen University Hospital

Blegdamsvej 9, 2100 Copenhagen, Denmark

Phone: +45 35453040; E-mail: marianne.benn@regionh.dk

Supplementary Table 1[28]

Dutch Lipid Clinic Network (DCLN) criteria		
**Family History**		**Score**
First degree relative with known premature coronary and/or vascular disease (Men <55 years, Women <60 years) **OR** First degree relative with known elevated LDL-cholesterol		1
**Clinical history**		
Patient with premature coronary artery disease (ages as above)		2
Patient with premature cerebral or peripheral vascular disease (ages as above)		or 1
**Physical examination**		
LDL-cholesterol	≥8.5 mmol/L	8
	6.5-8.4 mmol/L	or 5
	5.0-6.4 mmol/L	or 3
	4.0-4.9 mmol/L	or 1
DNA analysis - Functional mutation in the *LDLR, APOB* or *PCSK9* gene		or 8
**STRATIFICATION**		**Total Score**
**Definite FH**		>8
**Probable FH**		6-8
**Possible FH**		3-5
**Unlikely FH**		<3

Supplementary Table 2[28]

Simon Broome criteria
**Definite FH**
Total cholesterol >7.5 mmol/L **OR** LDL-cholesterol >4.9 mmol/L<br>**AND**<br>DNA analysis - Functional mutation in the *LDLR* or *APOB* gene
**Possible FH**
Total cholesterol >7.5mmol/L **OR** LDL-cholesterol >4.9 mmol/L<br>**AND**<br>First-degree relative with premature myocardial infarction **OR** with known elevated cholesterol levels

Supplementary Table 3[28]

Make Early Diagnosis to Prevent Early Death (MEDPED) criteria
**Probable FH**
Age 20-29 years **AND** [total cholesterol ≥7.5 mmol/L **OR** LDL-cholesterol ≥5.7 mmol/L]
Age 30-39 years **AND** [total cholesterol ≥8.8 mmol/L **OR** LDL-cholesterol ≥6.2 mmol/L]
Age ≥40 years **AND** [total cholesterol ≥9.3 mmol/L **OR** LDL-cholesterol ≥6.7 mmol/L]
